# Biomarker Report from the Phase II Lamotrigine Trial in Secondary Progressive MS – Neurofilament as a Surrogate of Disease Progression

**DOI:** 10.1371/journal.pone.0070019

**Published:** 2013-08-01

**Authors:** Sharmilee Gnanapavan, Donna Grant, Steve Morant, Julian Furby, Tom Hayton, Charlotte E. Teunissen, Valerio Leoni, Monica Marta, Robert Brenner, Jacqueline Palace, David H. Miller, Raj Kapoor, Gavin Giovannoni

**Affiliations:** 1 Department of Neuroinflammation, UCL Institute of Neurology, London, United Kingdom; 2 Independent Statistician, Haddenham, Bucks, United Kingdom; 3 Department of Clinical Chemistry, VU University Medical Center Amsterdam, Amsterdam, The Netherlands; 4 Laboratory of Clinical Pathology and Medical Genetics, Foundation IRCCS Neurology Institute “Carlo Besta”, Milano, Italy; 5 Blizzard Institute, Queen Mary University London, London, United Kingdom; 6 Department of Clinical Neuroscience, Royal Free Hospital, London, United Kingdom; 7 University Department of Clinical Neurology, Radcliffe Infirmary, Oxford, United Kingdom; University Hospital Basel, Switzerland

## Abstract

**Objective:**

Lamotrigine trial in SPMS was a randomised control trial to assess whether partial blockade of sodium channels has a neuroprotective effect. The current study was an additional study to investigate the value of neurofilament (NfH) and other biomarkers in predicting prognosis and/or response to treatment.

**Methods:**

SPMS patients who attended the NHNN or the Royal Free Hospital, UK, eligible for inclusion were invited to participate in the biomarker study. Primary outcome was whether lamotrigine would significantly reduce detectable serum NfH at 0-12, 12–24 and 0–24 months compared to placebo. Other serum/plasma and CSF biomarkers were also explored.

**Results:**

Treatment effect by comparing absolute changes in NfH between the lamotrigine and placebo group showed no difference, however based on serum lamotrigine adherence there was significant decline in NfH (NfH 12–24 months p = 0.043, Nfh 0–24 months p = 0.023). Serum NfH correlated with disability: walking times, 9-HPT (non-dominant hand), PASAT, z-score, MSIS-29 (psychological) and EDSS and MRI cerebral atrophy and MTR. Other biomarkers explored in this study were not found to be significantly associated, aside from that of plasma osteopontin.

**Conclusions:**

The relations between NfH and clinical scores of disability and MRI measures of atrophy and disease burden support NfH being a potential surrogate endpoint complementing MRI in neuroprotective trials and sample sizes for such trials are presented here. We did not observe a reduction in NfH levels between the Lamotrigine and placebo arms, however, the reduction in serum NfH levels based on lamotrigine adherence points to a possible neuroprotective effect of lamotrigine on axonal degeneration.

## Introduction

Sodium channel blockade is postulated to protect axons from anoxia-induced injury secondary to neuroinflammation. However, the Phase II lamotrigine trial in secondary progressive MS encountered an unexpected result with lamotrigine causing early cerebral volume loss that partially corrected after discontinuation of treatment. This effect may have been due in part to reversible fluid shifts or a reduction in inflammation due to the treatment.

Neurofilament proteins are the scaffolding proteins of neurons and are markers of neuro-axonal injury [1], [2]. CSF neurofilament (NfH) have been shown to be higher in all sub-types of MS compared to control subjects and elevated during the active [3] as well as the progressive phases correlating with clinical scales EDSS and MSFC [1], [4]. NfH is also detectable in the blood but has only been studied in one retrospective study evaluating response to interferon-β therapy, with no significant differences between the responders and the non-responders [5].

The biomarker study was to test whether lamotrigine could significantly reduce detectable NfH in sera at 12 and 24 months relative to baseline, compared to placebo. Other hypotheses explored included whether NfH predicts cerebral and spinal cord atrophy as measured using MRI and disability scales. A further exploratory panel of nitrite/nitrate, GFAP, BDNF and NGF NfL, ferritin, MBP, growth factors NCAM, GAP-43, *N*-acetylaspartate (NAA), cytokines CXCL12, BLC, CCL19, CCL21, TNFα, TNFβ, IFNγ, interleukins 2, 4, 5, 6, 8, 10, 12p70, 1b, oxysterols 24OHC/27OHC, lanosterol and cholesterol, DJ-1, osteopontin, were measured based on their evidence as biomarkers of disease activity in MS.

## Materials and Methods

### Study methodology

120 subjects with SPMS were recruited into the lamotrigine trial lasting two years (NCT00257855) [6]. Subjects for this study were recruited from the principle trial and thus the same inclusion/exclusion criteria applied: age 18–60 years, SPMS with a steady progression rather than relapse causing disability in the preceding two years, EDSS 4.0–6.5. Subjects with a rapid deterioration eligible for alternative treatment, taking Na^+^ or Ca^2+^ channel blockers, or had received immunosuppressive agents recently were excluded. This study was approved by the University College London Hospitals Committee and National Research Ethics Committee; written consent was obtained as outlined by the ethics committees.

**Figure 1:**
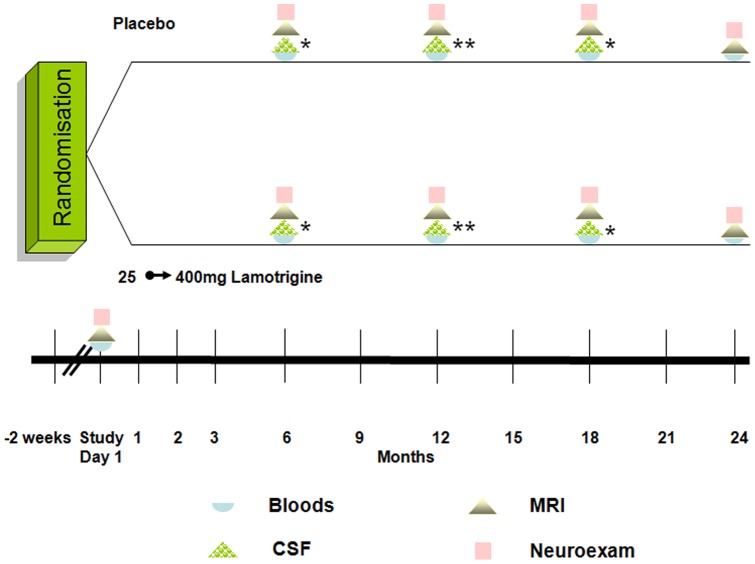
A diagrammatic representation of patient follow up and investigations. CSF sampling was performed at 6 and 18 m in 9 subjects and 12 m in 14 subjects.

101 subjects were included in the serum biomarker analyses owing to drop out or missing time points during the course of the study, and twenty-three underwent lumbar punctures. Lamotrigine group was further divided according to their adherence to treatment based on serum lamotrigine levels and tablet returns (see Table 1). Power calculations for the study were based on previous data using NfH, a sample size of 40 subjects in each arm was required to detect a difference of 30% in the proportion of subjects with undetectable NfH at 12 months (10% placebo *vs* 40% on lamotrigine). The sample size was based on a power of 80% at 5% significance level, with a combined loss to follow-up and/or non-compliance of 10%. Further modelling of serum biomarker data included all available samples (see Table S1). Cerebral volume, mean cervical spinal cord area, whole brain, grey matter and white matter magnetisation transfer ratios, and T1 and T2 lesions, EDSS and MSFC (25-foot timed walk, 9-hole peg test, and paced auditory serial addition test) were performed in all subjects. The timing of patient follow-up and investigations are shown in Figure 1.

**Table 1:** Summary characteristics of subjects in the study according to randomisation, serum adherence and tablet compliance (p>0.05).

	Lamotrigine group ITT	Placebo group ITT	Lamotrigine serum adherent	Lamotrigine non-serum adherent	Lamotrigine tablet compliant	Lamotrigine tablet non-compliant	CSF group
Number	48*†	53*†	24*	24*	28*	20*	23
Age, y (mean)	53	50	53	52	53	52	52
Progressive disease duration, y (mean)	8.4	8.6	7.8	8.9	7.7	9.2	10
EDSS (mean)	6.0	6.0	5.5	6.0	6.0	6.0	5.5
MSFC *z*-score (mean)	0.05	0.04	0.12	−0.01	0.09	0.01	−0.07

Three samples were present in each sub-group corresponding to time points 0–12–24 months *, and of the 120 subjects who participated in the biomarker study there were 19 drop-outs ^†^.

### Blood and CSF sampling

Blood was collected in Beckton-Dickinson (BD) tubes, centrifuged at 2,500 rpm for 10 minutes before aliquoting, CSF was centrifuged at 1,500 rpm for 5 minutes, cell-free CSF was aliquoted and stored at −80°C. The first aliquot of CSF and paired blood sample were processed for microbiology, protein and CSF/serum oligoclonal bands.

### Biomarker analysis

#### Serum

NfH (#NS170, Millipore; CV 4%, limit of detection/LOD 2 SD [standard deviations] above the background was 0.14 ng/ml), BDNF (#CYT306, Chemicon; CV 2%, LOD 0.27 ng/ml) and NGF (#CYT304, Chemicon; CV 2%, 0.23 ng/ml) were measured using ELISA kits. NOx was measured using vanadium assay by Rejdak *et al* [7] (CV 9%, LOD 0.99 μM/l). GFAP was measured using ELISA by Petzold *et al* [8] with modifications: primary antibody (#SMI-26R, Covance) at 1/1000, secondary polyclonal rabbit anti-GFAP (#Z033401, Dako) at 1/1000 and tertiary swine anti-rabbit HRP (#P0217, Dako) at 1/1000, with top standard of GFAP protein starting at 25 ng/ml (#345996, Calbiochem); CV 8%, LOD 0.22 ng/ml.

#### Paired CSF/serum/plasma

CSF 24OHC, 27OHC, cholesterol and plasma 24OHC and lanosterol (CV 5%, LOD 0.2 ng/ml) were measured by gas chromatography isotope-dilution mass spectrometry as in Leoni *et al* [9]. CSF and plasma NfL (CV 5%, LOD 0.09 ng/ml) was measured according to Kester *et al* [10]. NAA (CV 2%, LOD 0.01 μmol/l) was analysed by gas chromatography-mass spectrometry (GM-CSF) method by Jasperse *et al* [11]. CSF MBP was measured using ELISA kit (#T994ZB31, Cosmic Corporation; CV 11%, LOD 0.03 ng/ml). CSF ferritins, NCAM, GAP–43 were measured using in-house ELISA's (CV's 7, 5, and 7% respectively, and LOD 0.30 ng/ml, 12.43 ng/ml, and 8.73 ng/ml respectively). CSF and plasma GFAP and NfH were measured as above. Cytokines (IL12p70, IFN-γ, IL-2, IL-10, IL-8, IL-6, IL-4, IL-5, IL-1β, TNF-α, TNF- β) were measured flow cytometry using FlowCytomix kit (Bender Medsystems, CV 16%, LOD range 215.28 pg/ml to 1068 pg/ml depending on the cytokine). CCL21 (#D6C00, R? CV 11%, LOD 795.07 pg/ml), CXCL12 (#DSA00, R? CV 9%, LOD 69.74 pg/ml), BLC (DY801, R? CV 9%, LOD 4.91 pg/ml), and CCL19 (DY361, R? CV 4%, LOD 112.73 pg/ml) were measured using ELISA kits. Plasma osteopontin (OPN, #DOST00, R? CV 6%, LOD 0.61 ng/ml) and DJ-1 (#CY-9050, CircuLex; CV 5%, LOD 1.56 ng/ml) were measured using ELISA kits, and BDNF as above.

### Statistics

Changes in serum biomarkers NfH, NOx, GFAP, NGF and BDNF between 0–12 m, 12–24 m and 0–24 m were calculated for each patient. Differences in the distribution of changes between lamotrigine and placebo and lamotrigine adherent and non-adherent groups were determined by Kruskal-Wallis test. A comparison of subjects getting better (reduction in NfH, NOx and GFAP levels, and a rise in BDNF and NGF levels) were determined by Chi-square test. To assess the relationships between the serum biomarkers and the clinical and MRI measures the levels were categorised. For NfH and GFAP three categories were defined: absent (below assay detection), and above and below the median of the detectable concentrations. For NOx, BDNF and NGF four categories were defined bounded approximately by the quartiles of each biomarker's distribution (Table S1). Generalised linear models were used to assess the relationships between the categorised biomarkers and clinical and MRI measures. For each clinical and MRI measure, least squares means for each level of the biomarkers were obtained from the generalised linear model. These means and their confidence levels were tabulated and presented graphically. Walking times and 9-HPT times were log transformed as their distributions were more symmetrical on a log scale. Time to event analyses were conducted on walking times and 9-HPT times, in which uncompleted tests were treated as censored observations at 300 s. Survival curves of walking times and 9-HPT times were produced. T1 and T2 lesion volumes were also log transformed prior to analyses. All other clinical and MRI measures were analysed in their original units.

Screening for relationships between the 31 biomarkers in CSF and associated blood samples and clinical and MRI parameters were carried out by tabulating the rank correlations between every pair of variables. If a rank correlation suggested a statistically significant relationship (p<0.05), it was examined in further detail. Most serum immune markers were present in only few samples and we therefore constructed single flag variable indicating the presence of any serum immune biomarker.

## Results

### Treatment effect by comparing differences in NfH, NOx, GFAP, BDNF and NGF values between the lamotrigine and placebo groups

A summary table of serum biomarker values is in Table S1. There were no significant differences in the values of the five biomarkers between the two groups at 0–12 m, 12–24 m or 0–24 m (p>0.05).

### Treatment effect by comparing differences in NfH, NOx, GFAP, BDNF and NGF values based on lamotrigine serum adherence and tablet compliance

Reduction in NfH levels by measuring differences 0–12, 12–24 and 0–24 m between lamotrigine adherent and non-adherent groups revealed significant differences between the two groups at NfH 12–24 and NfH 0–24 m, p = 0.043 and p = 0.023, respectively. This was further supported by measuring rate of change, where lamotrigine adherent group showed a significant change between 0–24 m in NfH levels (p = 0.041), with NfH levels at 12–24 m having a p-value of 0.055. Grouping by improvement in biomarker values (i.e. reduction in NfH) was significant in NfH 0–24 m (p = 0.042). NOx, GFAP, BDNF and NGF values did not differ between the two groups over time (p>0.05). Analysis based on tablet returns did not reveal a significant difference in the serum biomarker levels, except based on grouping by improvement in biomarker levels for NfH 0–24 m (p = 0.012). However, seven subjects who had been considered tablet compliant had neither detectable serum lamotrigine levels or only at 6 m.

### Relationship between NfH, NOx, GFAP, BDNF and NGF values and clinical outcomes

**Figure 2:**
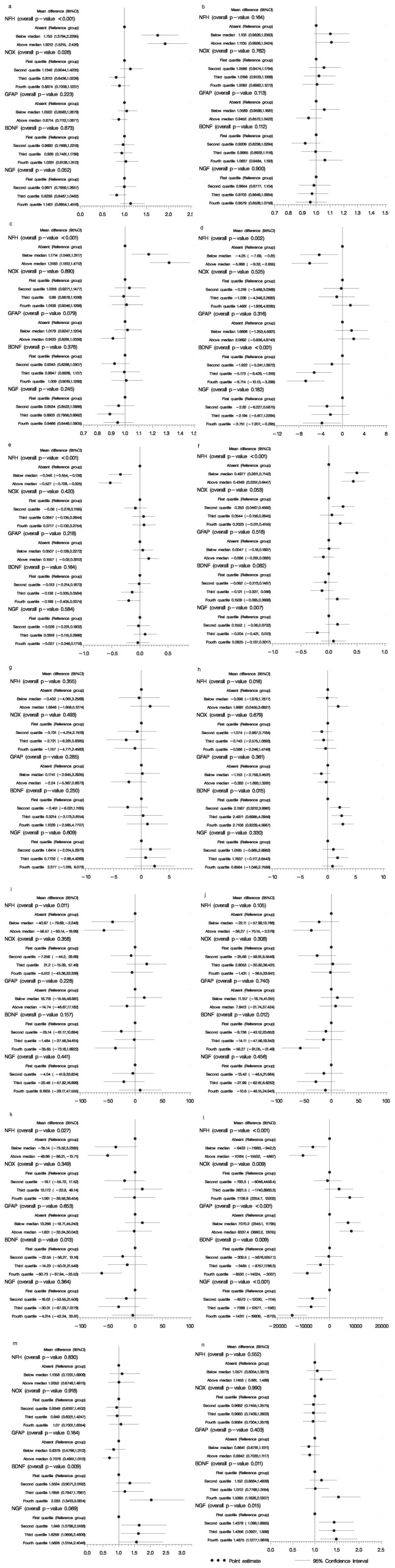
The relationship between serum biomarkers NfH, NOx, GFAP, BDNF, NGF and clinical (MSFC, EDSS, MSIS-29) as well as MRI measures are presented in tabular form and as forest plots. a) 25-foot walk (secs); b) 9-hole peg test, dominant had (secs); c) 9-hole peg test, non-dominant hand (secs); d) Paced Auditory Serial Addition Test (PASAT); e) Z-score; f) Expanded Disability Status Scale (EDSS); g) Multiple Sclerosis Impact Scale (MSIS-29) physical; h) MSIS-29 psychological; i) Magnetization Transfer Ratio (MTR) white matter; j) MTR grey matter; k) MTR whole brain; l) MRI Central Cerebral Volume (MRICCV); m) T1 volume; n) T2 volume.

**Figure 3:**
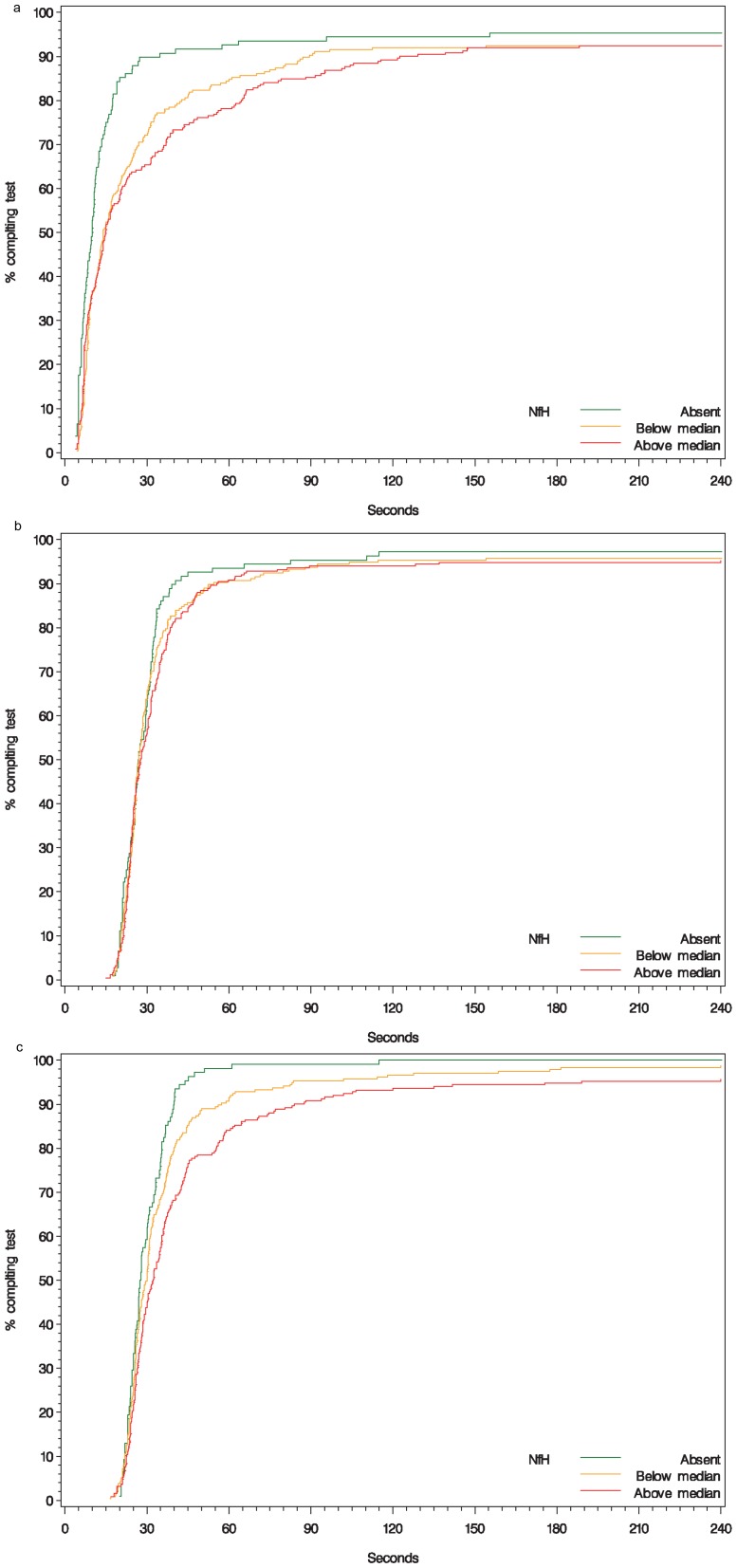
Survival curves of walking times and 9-hole peg test times (dominant and non-dominant hand) are plotted according to absent, below median or above median NfH levels.

NfH was found to be related to components of the MSFC. It was a strong predictor of walking times, with patients having any detectable NfH taking nearly twice as long to complete the test as those with none (p<0.001; Figure 2a). The 9-HPT times using the non-dominant hand were also longer in these patients, by 17% if NfH levels were present but below the median and by 32% if it was above the median (p<0.001; Figure 2c). This relationship between test times and NfH levels was confirmed by time to event analyses. Figure 3 shows the cumulative proportions of patients completing each test plotted against time. Results using the dominant hand revealed an increase of approximately 10% in 9-hole peg test times, although this was not found to be statistically significant (p>0.05; Figure 2b). The PASAT performance and z-score values decreased with increasing NfH levels (Figure 2d, e). NOx, GFAP and NGF were not found to be associated with the timed walk, 9-hole peg test time or z-scores. However, patients with high levels of NfH and BDNF had lower PASAT scores (Figure 2d).

EDSS was higher in patients with detectable NfH, and there was some indication of an association with NOx levels (Figure 2f). MSIS-29 psychological but not physical scores were found to be high in patients with higher BDNF levels, and there was some evidence that they were higher in patients with NfH levels above the median (Figure 2g, h). There was no correlation between EDSS and MSIS-29 physical scores, and the other serum biomarkers.

### Relationship between NfH, NOx, GFAP, BDNF and NGF values and MRI outcomes

Higher NfH was associated with lower MTR means in grey matter, white matter and whole brain (Figure 2i, j, k). BDNF in the highest quartile was also associated with lower MTR means, but there was no correlation with NOx, GFAP or NGF levels.

MRICCV was highly correlated with all five serum biomarkers (Figure 2l). It was higher in patients with higher levels of NOx and GFAP and lower in patients with higher levels of NfH, BDNF and NGF. There was no clear correlation between SCCA and any biomarker (not shown).

T1 and T2 lesion volumes were higher in patients with higher levels of BDNF and NGF (Figure 2 m, n).

### Relationship between the CSF and blood biomarkers and clinical outcomes

CSF biomarker analysis are summarised in Table S1. The rank correlations are shown in Table S2. Although many of these correlations were statistically significant, examination of the scatter plots did not demonstrate a consistent trend aside from plasma osteopontin. Higher plasma osteopontin values were associated with lower MSFC z-score, MRICCV, and grey matter and whole brain MTR (Figure S1). Non-parametric two sample tests based on the presence or absence of any serum markers of immune system activation also failed to reveal a difference for any clinical parameter.

## Discussion

We report on the prognostic value of serum neurofilament measurement in neuroprotective clinical trials. The primary hypothesis that lamotrigine will significantly reduce the proportion of cases with detectable NfH in sera at 12 and 24 months relative to baseline, compared to placebo was not evident in the intention to treat group, although the true effect of Lamotrigine may have been diluted by the low levels of compliance (40–50% non-adherence based on return of tablets and serum lamotrigine concentrations). Per protocol analysis based on serum and tablet adherence was therefore also performed, which also tested the pure pharmaceutical effect of the treatment. There was significant reduction in serum NfH of subjects with detectable serum lamotrigine between 12–24 m and 0–24 m, suggesting a possible neuroprotective effect on axons by lamotrigine. The delay in the reduction of NfH by 12 months implies a lead-lag effect between taking the drug and neuroprotection. Lim *et al*. [12] demonstrated that elevation in NfH between 0–3 weeks was predictive of poor clinical outcome at week 8. We did not see a difference in serum biomarker levels based on intention to treat analysis, nor did we note a difference based on tablet returns; apart from NfH levels between 0–24 m when comparing percentage of subjects “getting better” on lamotrigine. Using tablet returns as a way of assessing treatment compliance is prone to errors in judgment as demonstrated, whilst serum drug levels are probably the closest way to reliably assessing compliance. The original reports of this trial based on MRI cerebral volumes did not support a neuroprotective effect of lamotrigine, but this may have been confounded by fluctuations in parenchymal water content from sodium channel blockade, as indicated by partial improvement upon treatment discontinuation [6].

NfH was a strong predictor of ambulation and disability based on MSFC and EDSS, supporting the use of NfH as a surrogate measure of disability. This is confirmed by the time-to-event modelling for completion of the timed 25-foot walk. Interestingly, performance in the 9-HPT and its relationship with NfH levels was found predominantly in the non-dominant hand which may be related to the loss in manual dexterity that may be more obvious with the non-dominant hand as the test is normally performed faster using the dominant hand [13]. Others have also shown that high NfH levels correlate with poor visual outcome [14], [15]. Neuropsychological test scores from the PASAT and MSIS-29 appeared to be also responsive to changes in NfH, with patients with higher NfH having lower PASAT scores and higher MSIS-29 psychological scores, possibly related to the significant neurodegenerative changes observed in grey matter disease [16] or in relation to EDSS deterioration, respectively; McGuigan *et al*. found that MSIS-29 increased with EDSS deterioration [17].

NfH correlated well with all MRI-derived measures except for conventional T1 and T2 volumes. MRICCV and MTR were both lowered in patients with higher NfH, supporting earlier associations of NfH with clinical measures and connecting progressive brain atrophy in SPMS to NfH release and a reduction in cerebral volumes (MRICCV) and global disease burden (MTR). Our observed lack of correlation between spinal cord area and NfH levels may have been partly confounded by the lack of sensitivity of spinal cord MR imaging to axonal disease [18].

**Figure 4:**
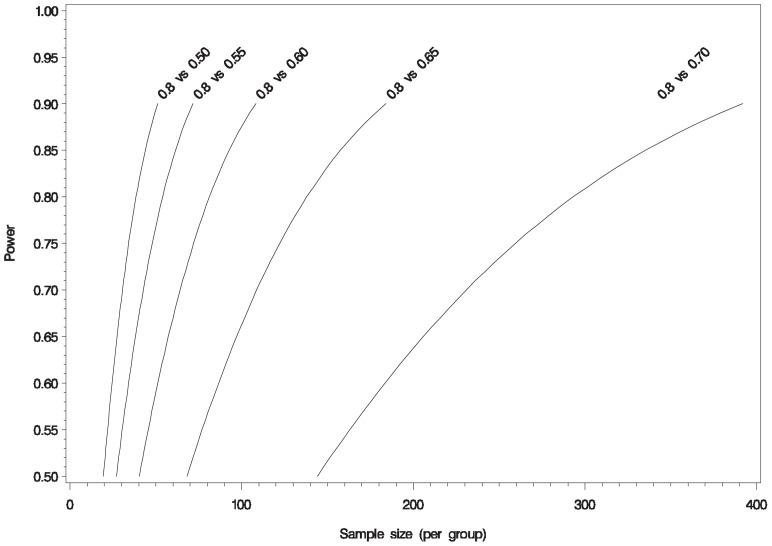
Power curves for determining sample size for changes percentage change in NfH levels.

Based on the parameters obtained for NfH we have estimated the sample sizes that future studies would need to detect changes in the proportion of patients with detectable NfH. In this study 80% of samples had detectable NfH and we calculated the sample sizes required to detect differences between this and 50, 55, 60, 65 and 70% in a comparator group (Figure 4).

We also analysed serum NOx, GFAP, BDNF and NGF, but found significant association was between walking times or 9-HPT times. However, MRI central cerebral volumes highly correlated with NOx and GFAP as expected since inflammation and oedema may increase brain volume. Cheriyan J *et al*. [19] noted transient increases in brain volume during periods of MRI gadolinium activity. Unexpectedly, high BDNF and NGF were associated with lower MRI CCV and higher T1 and T2 volumes, whilst BDNF levels in the highest quartile were associated with lower MTR means. This can be interpreted as either reactive neuroplasticity with upregulation of neurotrophins following injury, or as markers of inflammation, as both have been shown to play a regulatory role in inflammation [20]. Similarly, patients with higher BDNF, but not NGF levels were found to have lower PASAT scores. More work is needed in order to better understand the functional and clinical implications of these findings.

Of the 31 paired CSF/serum/plasma biomarkers studied, no clear trends emerged from the analyses aside from plasma osteopontin (Figure S1). Higher levels of osteopontin have previously been associated with greater disability, and our analysis supports this with subjects exhibiting z-scores below average on the MSFC, as well as reduced MRICCV and grey matter MTR values [21]. The lack of association with the other biomarkers is most likely secondary to the low statistical power. Correction for multiple comparisons was not performed at this stage as the work is exploratory in nature and positive findings should be tested in future prospective studies. However, as a collaborative study with John Hopkins University the CSF samples were analysed using mass spectrometry based on serum drug adherence [22], which revealed a significant reduction in 11 protein biomarkers, including neurofilament.

In conclusion, the results of this study failed to show any benefit of lamotrigine in the prevention of axonal breakdown by lowering NfH levels compared to the placebo arm. A further subgroup analysis based on lamotrigine adherence, however, suggests that it may have a beneficial effect on axonal protection and it would be premature to abandon this hypothesis in entirety. We have demonstrated that NfH is very good surrogate marker of neurodegeneration and correlates well with clinical and MRI biomarkers of progression. The sample size calculations provided in this paper should pave the way for neuroprotection trials using serum NfH.

## Supporting Information

Figure S1 **Plasma osteopontin and its relationship with the z-score, MRICCV, MTR grey matter and whole brain are presented.**
(TIF)

Table S1 **Biomarker values in serum, plasma and CSF samples from the lamotrigine trial.** They are grouped approximately by the quartiles of each biomarker's distribution, and in analysis where only a few were detectable as absolute values.
(TIF)

Table S2 **Rank correlation coefficients between biomarkers and test scores (a) and MRI measures.** Statistically significant correlations (absolute values >0.346) are shaded, red for positive correlations and green for negative.
(TIF)
