# Supplementary figures and images for: Biomarker Report from the Phase II Lamotrigine Trial in Secondary Progressive MS – Neurofilament as a Surrogate of Disease Progression
Source: PLoS One. 2013 Aug 1;8(8):e70019. doi: 10.1371/journal.pone.0070019 (PMC3731296; doi:10.1371/journal.pone.0070019)

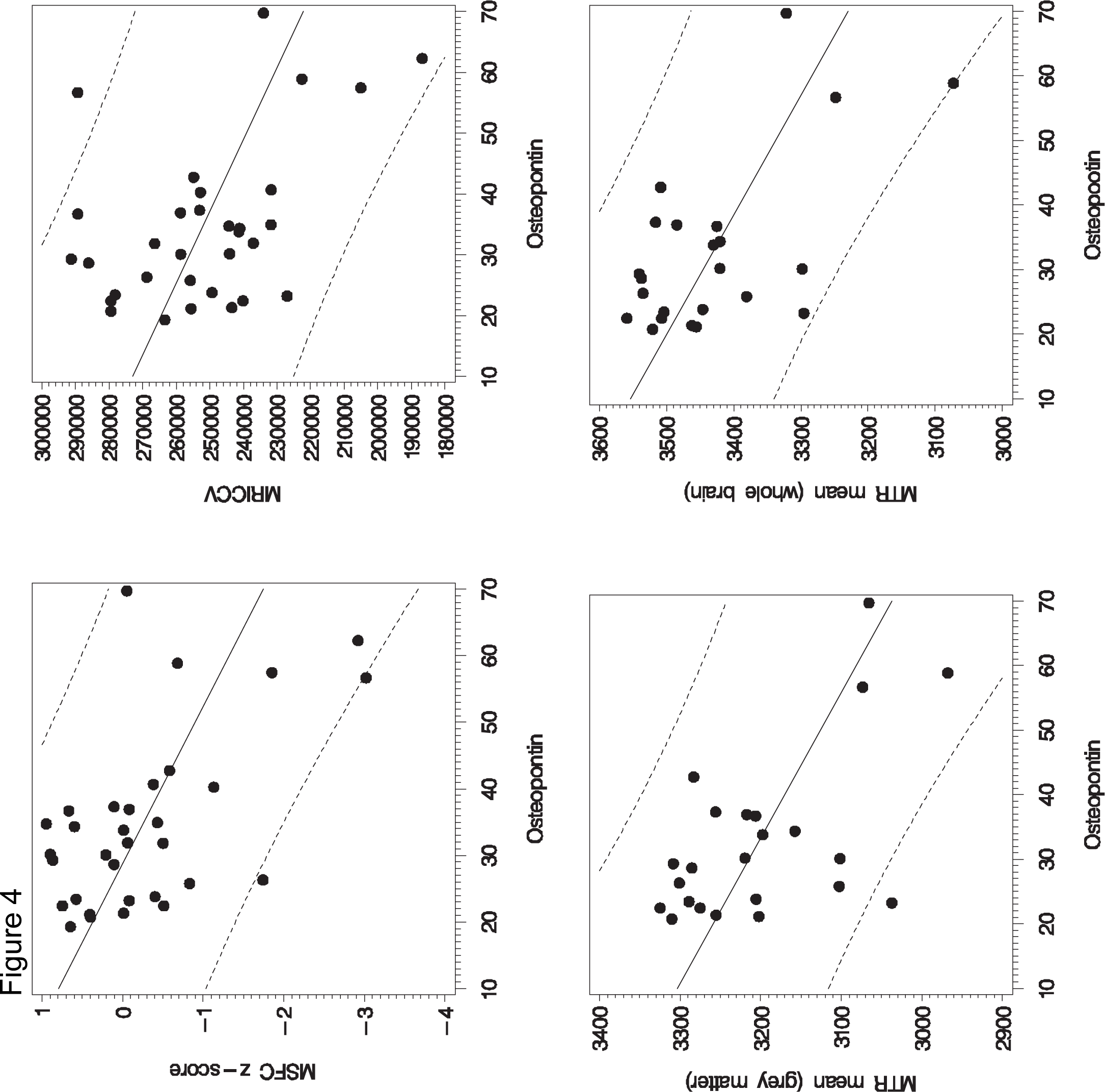

Supplement: Figure S1 — Plasma osteopontin and its relationship with the z-score, MRICCV, MTR grey matter and whole brain are presented. (TIF) [file pone.0070019.s001.tif]

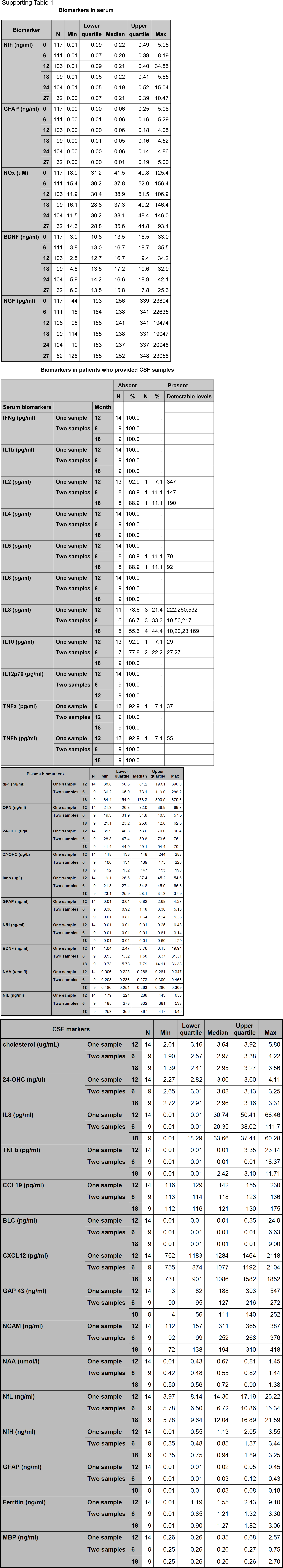

Supplement: Table S1 — Biomarker values in serum, plasma and CSF samples from the lamotrigine trial. They are grouped approximately by the quartiles of each biomarker's distribution, and in analysis where only a few were detectable as absolute values. (TIF) [file pone.0070019.s002.tif]

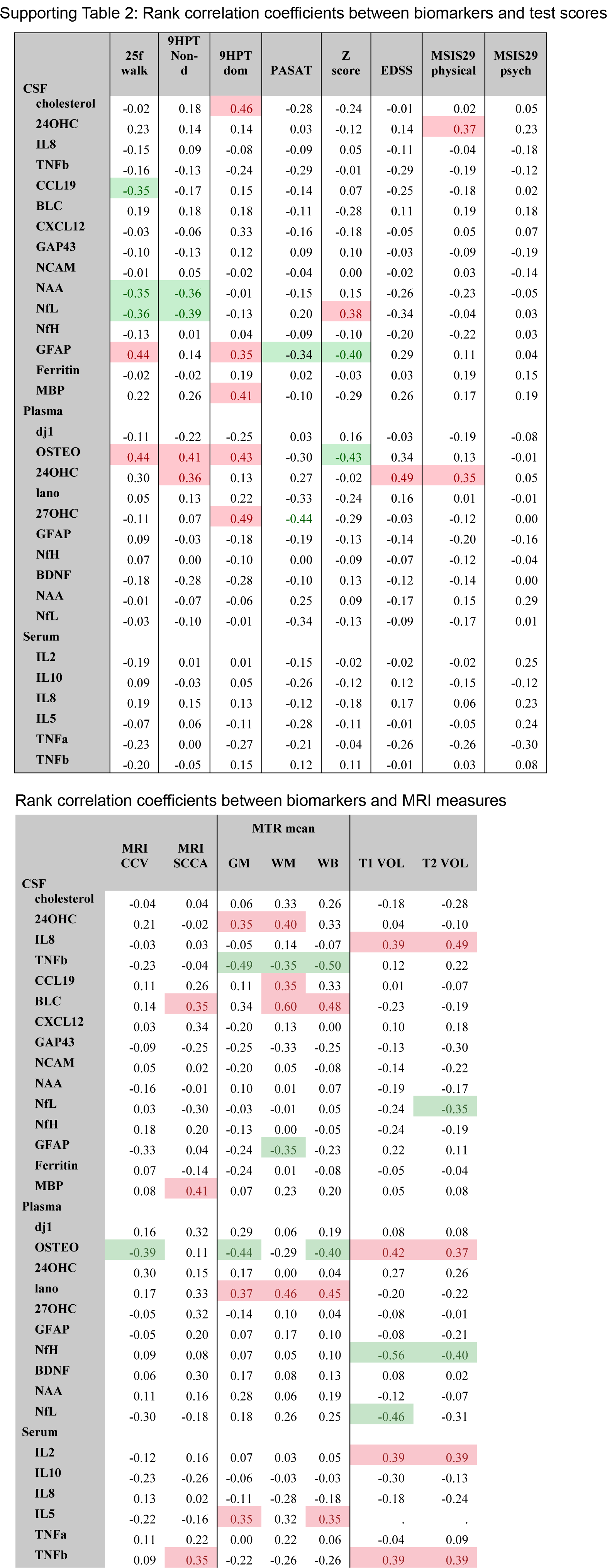

Supplement: Table S2 — Rank correlation coefficients between biomarkers and test scores (a) and MRI measures. Statistically significant correlations (absolute values >0.346) are shaded, red for positive correlations and green for negative. (TIF) [file pone.0070019.s003.tif]
